# The meiotic cohesin subunit REC8 contributes to multigenic adaptive evolution of autopolyploid meiosis in *Arabidopsis arenosa*

**DOI:** 10.1371/journal.pgen.1010304

**Published:** 2022-07-13

**Authors:** Chris Morgan, Emilie Knight, Kirsten Bomblies

**Affiliations:** 1 John Innes Centre, Norwich, United Kingdom; 2 Plant Evolutionary Genetics, Institute of Plant Molecular Biology, Department of Biology, ETH Zürich, Zürich, Switzerland; The University of North Carolina at Chapel Hill, UNITED STATES

## Abstract

Genome duplication, which leads to polyploidy, poses challenges to the meiotic segregation of the now-multiple homologous chromosome copies. Genome scan data showed previously that adaptation to polyploid meiosis in autotetraploid *Arabidopsis arenosa* is likely multigenic, involving genes encoding interacting proteins. But what does this really mean? Functional follow-up studies to genome scans for multigenic traits remain rare in most systems, and thus many mysteries remain about the “functional architecture” of polygenic adaptations. Do different genes all contribute subtle and additive progression towards a fitness optimum, or are there more complex interactions? We previously showed that derived alleles of genes encoding two interacting meiotic axis proteins (ASY1 and ASY3) have additive functional consequences for meiotic adaptation. Here we study derived versus ancestral alleles of the meiotic cohesin subunit REC8, which has roles in chromatin condensation, recruiting the axes, and other critical functions in meiosis. We use genetic and cytological approaches to assess the functional effects of REC8 diploid versus tetraploid alleles, as well as their interaction with ancestral versus derived alleles of ASY1 and ASY3. We show that homozygotes for derived (tetraploid) REC8 alleles have significantly fewer unpaired univalents, a common problem in neotetraploids. Interactions with ASY1 and ASY3 are complex, with the genes in some cases affecting distinct traits, and additive or even antagonistic effects on others. These findings suggest that the road to meiotic adaptation in *A*. *arenosa* was perhaps neither straight nor smooth.

## Author summary

Genome duplication, which results in polyploidy, is fairly common in eukaryotes, especially plants, and has been linked to adaptation and speciation in nature, as well as increased yield and stress resilience in crops. Yet, newly formed polyploids face many challenges. To understand more fully how polyploids evolve and adapt, or to more efficiently utilize them in crop improvement, it is important that we learn what it really takes to be polyploid. As part of a longer-term effort to characterizing the multigenic adaptations to polyploid chromosome segregation, here we functionally characterize the effect in meiosis of ancestral versus derived alleles of one gene under selection in a polyploid, as well as its interactions with two other genes. The at-times complex interactions we discover provide insights into what seems to have been a sometimes-rocky path to multigenic adaptation.

## Introduction

Our understanding of the genetic architecture of adaptation has benefitted greatly from improvements in whole genome sequencing, which have made it possible to undertake so-called “genome scans” for selection. This ability, in turn, opens the opportunity to study the genetic basis of adaptation from a “reverse genetics” perspective, where we start from identifying genes with signatures of selection, and use these to try to understand what traits they affect and why they might have been important in adaptation [1–3]. This reverse approach complements the forward phenotype-based approach, and has the potential to provide novel insights into the molecular basis of adaptation, especially for non-obvious, or multigenic traits. Nevertheless, challenges remain [4], and functional follow-up to test the effects of the genes with evidence of selection remain rare in most systems, especially for multigenic traits, which are particularly laborious and high-risk. We know from theory and empirical studies that multigenic adaptation should be common [5–9], a notion which genome scan data, including our own [10–12], generally support. However, while genome scans have already provided insights into the genetic architecture of multigenic adaptation, we know comparatively less about what can be thought of as the “functional architecture” of multigenic adaptation. How strong are the effects of individual loci when multiple loci are under selection? Do selected alleles act additively, synergistically, or even antagonistically? Do they have overlapping pleiotropic functions, or do they contribute independently to different aspects of adaptive traits?

Over the last decade, *Arabidopsis arenosa* has emerged as a model organism for studying the molecular basis of adaptation (e.g. [13,14]), among other things to whole genome duplication (WGD), which gives rise to polyploidy [10–12,15–17]. Genome scans to investigate adaptation to polyploidy have provided evidence that multiple genes encoding meiosis proteins are among the loci showing the strongest evidence of selection in the polyploid *A*. *arenosa* lineage [10–12,17]. This is hypothesized to reflect the fact that WGD poses a serious threat to fertility and genome integrity, by presenting novel challenges to chromosome pairing and segregation during meiosis [18–20].

The challenges polyploids face in meiotic segregation of the additional chromosome copies may be particularly acute in autopolyploids, which are formed from within-species WGD, and thus possess multiple, equally similar homologous copies of each chromosome [21,22]. During diploid meiosis, the formation of crossovers (COs) between pairs of homologous chromosomes is essential for promoting the stable segregation of homologs during anaphase I, as well as for introducing genetic diversity within offspring [23]. In most organisms, CO maturation is facilitated by formation of the meiotic axis and synaptonemal complex, proteinaceous structures that organise chromosomes into threadlike arrays of chromatin loops and synapse homologous axes together along their length, respectively [24,25]. In autopolyploids, due to the presence of more than two copies of each homolog, synapsis and subsequent CO formation can occur between multiple homologs simultaneously, creating linkages called multivalents. These structures are associated with an increased risk of chromosome mis-segregation and can lead to the formation of unbalanced, or even inviable gametes [20,22]. In a recent study, we found that polyploid meiotic stabilization is likely attributable, at least in part, to a strengthening of crossover interference or an increase in its efficiency of propagation along the chromosomes, which in turn helps reduce the number of multivalents and unpaired univalents [16]. We hypothesized that this could result from stiffening of axial element structures, which may explain why the meiotic axis proteins ASY1 and ASY3 show evidence of selection in *A*. *arenosa* autotetraploids [10,11]. We also found that established polyploids had shorter synaptonemal complexes and fewer crossovers than neopolyploids; likely all of these features function together to stabilize polyploid chromosome pairing and segregation [16].

The genome scans done for adaptation to WGD in *A*. *arenosa* demonstrated that at least eight essential meiosis genes are under strong selection in naturally established populations of autotetraploid *A*. *arenosa*, suggesting that meiotic adaptation in the polyploid lineage is an example of multigenic adaptation [10,11]. Of the multiple meiotic genes putatively under selection in the tetraploid, all are known to encode proteins that directly or indirectly interact, and are all known from mutant studies in other species to regulate related processes relevant to chromosome pairing and segregation that clearly represent challenges for polyploid meiosis. We already showed that derived alleles for genes encoding two meiotic axis proteins, ASY1 and ASY3 (homologs of Hop1 and Red1 in *S*. *cerevisiae*), affect multiple traits associated with tetraploid meiotic stability, such as reduced multivalent frequency and reduced axis length [15]. The derived alleles of ASY1 and ASY3 have primarily additive effects, though ASY1 has a generally stronger effect than ASY3 [15]. This additivity is perhaps unsurprising, given that ASY1 and ASY3 are directly-interacting critical components of the chromosome axis [26,27]. What the derived alleles of the remaining genes showing evidence of selection do, and whether or not they also contribute additively to the same phenotypes, remained untested.

In this study, we continue the task of understanding this multigenic adaptation by investigating the functional role of the derived (tetraploid-specific) allele of the meiotic cohesin subunit REC8 in the stabilisation of autotetraploid meiosis. This is motivated by the fact that REC8 is an essential component of meiosis known to affect relevant traits like axis assembly, axis / synaptonemal complex length and crossover frequency [16–19,28–31]. Moreover, REC8 is known to directly interact with the axis components and to recruit them to the chromosomes [32]. We also previously showed that REC8 might have been one of the oldest selective sweeps in tetraploid *A*. *arenosa*, likely predating selection on ASY3, and maybe even ASY1 [12]. This makes it especially interesting to understand its functional effects as it might have been a “frontline” player in the early adaptation of the polyploid lineage. We bred established tetraploid lines of *A*. *arenosa* that are homozygous for the ancestral, diploid alleles of *REC8* in an otherwise tetraploid background. Since REC8 interacts with the axis proteins to ensure correct formation of the meiotic axis, we also studied the genetic interaction of derived alleles of *REC8* with those of *ASY1* and *ASY3*.

We find that one of the strongest associations with the derived, tetraploid allele of REC8 is a reduced frequency of undesirable metaphase I univalents, which neotetraploids have significantly more of than evolved tetraploids do [16]. Otherwise, we find generally subtle quantitative effects on several other meiotic traits. Interactions between the ancestral vs. derived alleles of REC8 with the ancestral vs. derived alleles of axis proteins are complex. Sometimes their effects are independent, whilst for other traits they are additive, and even sometimes antagonistic. Based on our findings, we propose a multi-step, multi-gene model that may explain the evolution of enhanced tetraploid meiotic stability in *A*. *arenosa*, in which the derived allele of REC8 may have been selected initially to reduce univalent frequency. The subsequent evolution of derived alleles at ASY1 and then ASY3 modified additional traits, including reducing multivalent rate, and have both additive and antagonistic interactions with the derived allele of REC8. We also recognize an alternate possibility, namely that REC8 may be under selection primarily to maintain interactions with other proteins that are evolving functional novelty.

## Results

### The effect of ancestral vs. derived alleles of REC8 on tetraploid metaphase I phenotypes

To test the effects of ancestral, diploid (D) versus derived, tetraploid (T) alleles of REC8 on meiotic stability in tetraploid *A*. *arenosa*, we first generated lines that were homozygous for either the D or T alleles. We did this as described previously, by taking advantage of the fact that D alleles of some genes persist within one lineage of autotetraploid *A*. *arenosa* due to gene flow from diploid populations [11,33,34]. Using PCR-based markers, we identified plants from the tetraploid KOWA population [35] that were heterozygous for T and D alleles of REC8. We intercrossed these plants to generate lines that were either homozygous for the T allele (REC8 TTTT), homozygous for the D allele (REC8 DDDD) or heterozygous (REC8 TxD) in an otherwise tetraploid background (S1 Fig). Note, heterozygotes are referred to as “TxD” because the genotyping approach used did not allow us to confidently differentiate between heterozygotes with different copy numbers of the T or D alleles (TDDD, TTDD, or TTTD plants).

**Fig 1:**
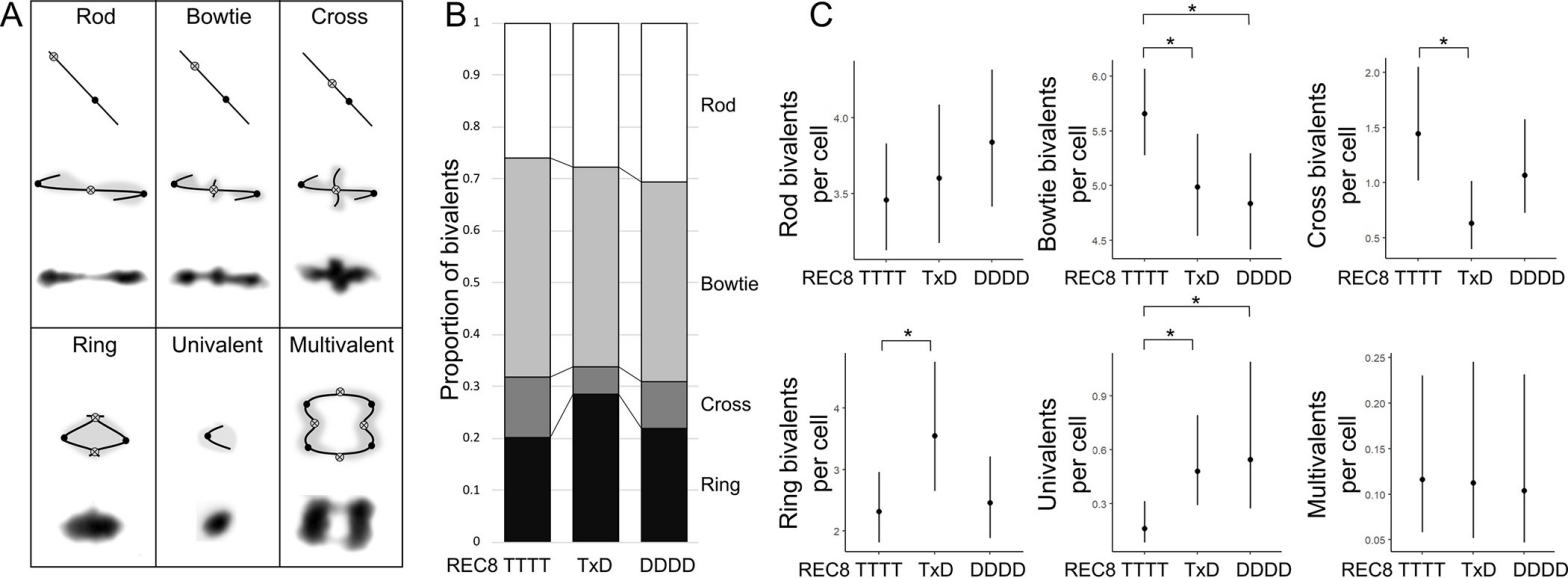
Analysis of REC8 TTTT, TxD and DDDD metaphase I cells. **(A)** Key explaining the shapes of different metaphase I chromosomal configurations. Example images of each metaphase configuration (stained with DAPI) are shown (bottom, each box) alongside a stick interpretation, with chromatin shaded in grey (middle) and a cartoon indicating the predicted centromere (black circle) and CO (open circle with cross) position on recombining chromosomes. **(B)** Stacked bar chart showing the mean proportional frequency of different bivalent shapes in REC8 TTTT, TxD and DDDD metaphase I cells. **(C)** Plots showing the number of different chromosomal configurations per cell in REC8 TTTT, TxD and DDDD metaphase I cells. Dots indicate trait means and error bars 95% confidence intervals calculated from GLMM models. Significant between genotype *p* values are indicated: * *p* < 0.05.

To assess meiotic stability in REC8 TTTT, REC8 TxD and REC8 DDDD plants, we first performed cytological analysis of DAPI-stained metaphase I spreads as we did previously for ASY1 and ASY3 [15]. We emphasise that this analysis was only performed on male meiocytes (which are experimentally more tractable for cytological analysis) and therefore our results and conclusions are only directly relevant to male meiosis. Metaphase I images from each genotype were scored blind for the frequency of different bivalent shapes (rod, bowtie, cross and ring) as well as for the presence of abnormal multivalent configurations or unpaired (univalent) chromosomes (Fig 1A). Bivalents with different shapes may arise from differences in CO number and positioning [36], though while likely accurate for CO number, they may not always be reliable for CO position (see:[15,37]). In general, “rod” bivalents are interpreted as possessing a single distal CO (close to a chromosome end), “bowtie” bivalents as possessing a single interstitial CO, “cross” bivalents possessing a single proximal CO (close to the centromere), and “ring” bivalents possessing at least two COs (one on each chromosome arm). Multivalent chromosomes arise when 3 or 4 chromosomes are linked simultaneously by COs, while univalents are formed when a chromosome fails to form a single CO.

We only included images that were of sufficient quality that at least 10 of the 16 possible chromosome pairs could be reliably scored. The presence of overlapping or partially-spread chromosomes prevents us from confidently scoring all chromosomes within the majority of metaphase I cells, thus this cut-off that was chosen prior to data analysis in our prior study of ASY1 and ASY3 [15], as this cutoff provided a good compromise between high quality spreads, and yet having sufficient numbers (both of chromosomes and images). Moroever, since it was the same cut-off as previously used, it increases comparability among results [15]. In total, our final dataset included 186 cells from 10 REC8 TTTT plants, 110 cells from 6 REC8 TxD plants and 115 cells from 9 REC8 DDDD plants (S1 Table). We then used generalized linear mixed models (GLMM), including genotype as a fixed effect and plant as a random effect, to test for statistical differences in the frequency of different metaphase I chromosomal configurations in different genotypes (Fig 1B and 1C). GLMMs have been used previously for this type of analysis, as they are well suited for analysing count data, and they can account for biological variation by the inclusion of biological replicates (plants) as a random factor within the models, preventing the introduction of type I errors by sample pseudoreplication [15]. In addition to our counts-per-cell GLMM analysis (Fig 1C), we also performed GLMM analysis on count data normalised against the total number of scorable chromosomes in each cell (S2 Fig) and Kruskal-Wallis tests on data pooled from all plants of each genotype (S2 Table) to further confirm significant between-genotype differences identified in the counts-per-cell GLMM analyses.

We identified several differences in the frequency of metaphase I chromosomal configurations in the different genotypes (Fig 1B and 1C). With respect to bivalent shape, we found that REC8 TTTT plants had significantly more bowtie bivalents per cell than either the REC8 DDDD (Poisson-GLMM, 5.66 95% CI [5.28, 6.06] vs. 4.83 95% CI [4.42, 5.29], *p* = 0.0072) or REC8 TxD plants (Poisson-GLMM, 5.66 95% CI [5.28, 6.06] vs. 4.99 95% CI [4.54, 5.47], *p* = 0.033). We also found cases where heterozygotes have more extreme phenotypes than either homozygote: REC8 TxD plants have significantly fewer cross bivalents than REC8 TTTT plants (Poisson-GLMM, 0.63, 95% CI [0.396, 1.01], vs. 1.44, 95% CI [1.018, 2.05], *p* = 0.0057) and significantly more ring bivalents than REC8 TTTT plants (Poisson-GLMM, 3.55, 95% CI [2.65, 4.75] vs. 2.32, 95% CI [1.81, 2.96], *p* = 0.028). Why the heterozygotes have extreme values for these traits is unclear, but parallels previous findings for ASY1 [15]. Finally, we also found that REC8 TTTT plants had significantly fewer univalent chromosomes per cell compared with REC8 DDDD plants (Poisson-GLMM, 0.16 95% CI [0.082, 0.31] vs. 0.55 95% CI [0.27, 1.09], *p* = 0.012) or REC8 TxD plants (Poisson-GLMM, 0.16 95% CI [0.082, 0.31] vs. 0.48 95% CI [0.29, 0.79], *p* = 0.0086. Note, the Kruskal-Wallis test for this one comparison was not significant, S2 Table). This parallels the previous finding that neotetraploids (carrying D alleles of all meiosis genes) have increased univalent frequencies relative to evolved tetraploids [16]. REC8 genotype showed no significant effect on multivalent frequency, another polyploid meiotic stabilization trait.

### Metaphase I phenotypes are preceded by REC8 genotype-specific differences in pachytene cells

Chromosome pairing and CO-designation, which affect metaphase configurations, occur before metaphase I, during the pachytene substage of meiotic prophase I [23,25,38]. Hence, we investigated CO patterning and chromosome behaviour in more detail in *A*. *arenosa* pachytene cells using a combination of immunocytochemistry and super-resolution microscopy.

**Fig 2:**
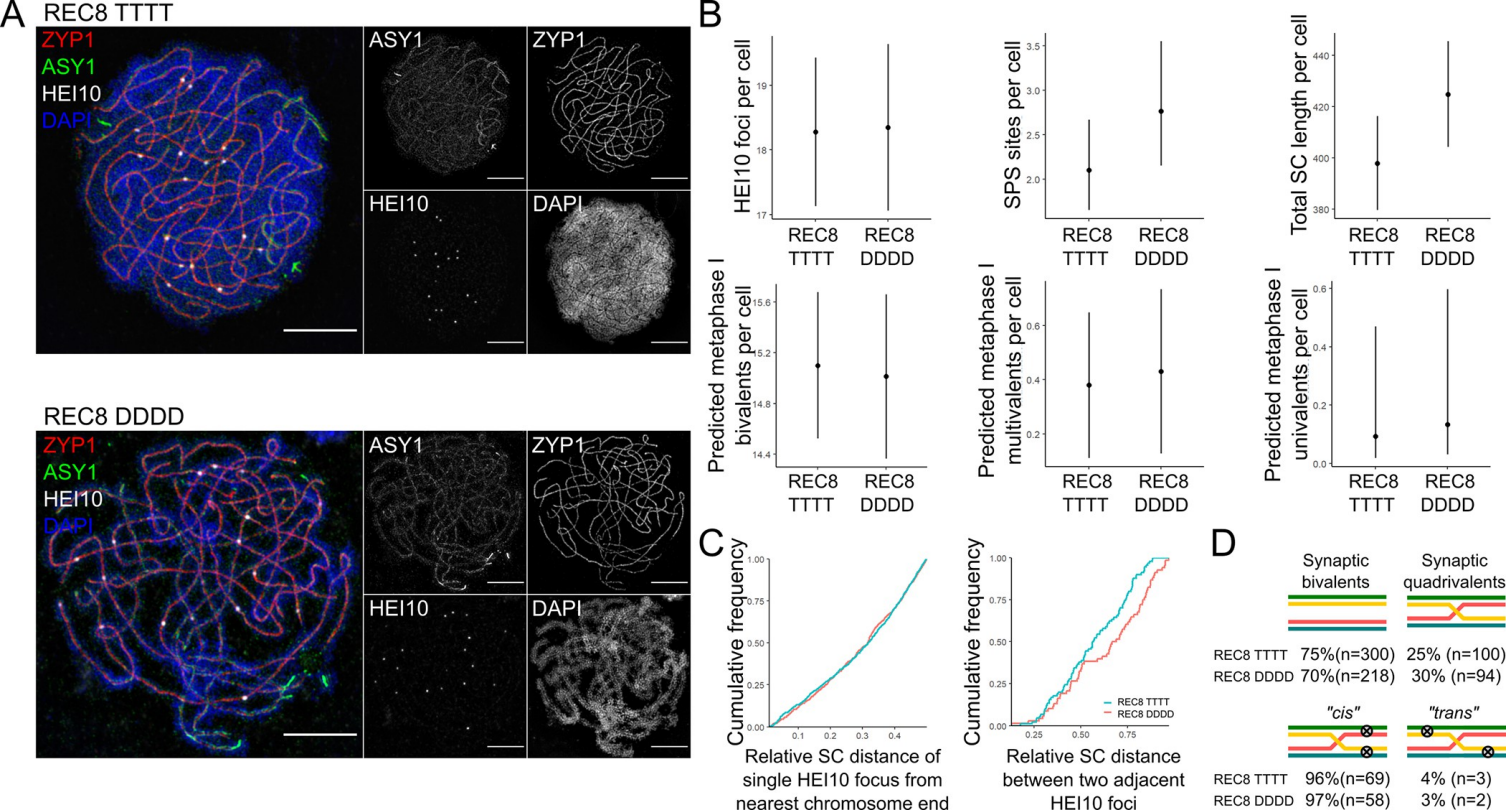
Analysis of REC8 TTTT and DDDD late-pachytene cells. **(A)** Example images of REC8 TTTT (top) and REC8 DDDD (bottom) late-pachytene cells imaged using 3D-SIM and labelled for ZYP1 (red), ASY1 (green), HEI10 (grey) and DAPI (blue). Maximum intensity projections of 3D images are presented. Scale bar = 5 μm. **(B)** Top row—plots showing the number of late-HEI10 foci (left), number of SPS sites (middle) and total SC length in μm (right) per cell in REC8 TTTT and DDDD late-pachytene cells. Bottom row—plots showing the predicted number of metaphase I bivalents (left), multivalents (middle) and univalents (right) per cell based on the relative position of late-HEI10 foci and SPS sites along pachytene chromosomes in REC8 TTTT and DDDD cells. Dots indicate trait means and error bars 95% confidence intervals calculated from GLMM models. **(C)** Cumulative frequency plots showing the distance of single late-HEI10 foci from the nearest chromosome end (left) and the distance between two late-HEI10 foci (right) in units of relative SC length in REC8 TTTT (blue) and REC8 DDDD (red) late-pachytene cells. **(D)** Frequency of different synaptic and crossover outcomes.

We imaged late-pachytene REC8 TTTT and REC8 DDDD cells labelled for ZYP1, HEI10, ASY1 and DAPI using 3D-SIM microscopy (Fig 2A). ZYP1 is a component of the synaptonemal complex [39] and detecting it allowed us to identify, segment and measure each synaptic bivalent or synaptic quadrivalent in each pachytene cell using the Simple Neurite Tracer plugin to ImageJ [40]. ASY1 is a component of the meiotic axis [27] and binds with greater intensity to unsynapsed regions of the axis, allowing us to quantify the extent of asynapsis and highlight the presence of synaptic partner switch (SPS) sites within cells, which are also often flanked by regions of increased ASY1 intensity [16]. SPS sites are regions where homologs exchange their synaptic partner, switching from one homolog to synapse with one of the other two available homologs, a situation unique to polyploids, much more common in neopolyploids than evolved polyploids, and positively correlated with metaphase I multivalent frequency [16]. HEI10 is a marker of CO-designated sites in late-pachytene cells [41]. Imaging this selection of proteins allowed us to quantify SC length, CO position, CO frequency, SPS site position and SPS site frequency along each component chromosome in each cell, all of which are meiotic phenotypes that differ between neo- and established polyploids (Figs 2B and S3; [16]). We also used this information to predict the expected metaphase I outcome (bivalent, univalent or multivalent) for each set of four homologs by comparing the relative positions of CO-designated sites and SPS sites (Figs 2B and S3) [16].

In total we imaged 50 cells from 5 REC8 TTTT plants and 39 cells from 4 REC8 DDDD plants (S3 Table). Between-genotype statistical differences in CO frequency, SPS frequency and SC length were calculated using GLMMs (Fig 2B). We found no statistically significant differences between REC8 TTTT and REC8 DDDD genotypes, however there were clear trends. We use the term ‘trend’ in this study to describe inferences made from the directionality or magnitude of consistent, yet statistically non-significant differences (p-values between 0.05 and 0.15) within our sample data. We found that REC8 TTTT cells tend to have fewer SPS sites per cell than REC8 DDDD cells (Poisson-GLMM, 2.10, 95% CI [1.66, 2.66] vs. 2.76, 95% CI [2.16, 3.54], *p* = 0.112) and shorter SC length (LMM, 398 μm, 95% CI [376, 420] vs. 425 μm, 95% CI [400, 449], *p* = 0.094). No significant differences were detected in the frequency of predicted metaphase I outcomes between different genotypes, with REC8 TTTT cells having only marginally fewer predicted univalent / trivalent associations per cell than REC8 DDDD cells (Poisson-GLMM, 0.092, 95% CI [0.018, 0.459] vs. 0.133, 95% CI [0.0305, 0.584], *p* = 0.712). This latter observation suggests the failures that cause univalency in metaphase I may primarily arise from defects later in meiotic progression than observed here.

To assay CO positioning we pooled measurements from all bivalents of each genotype, and distributions were compared using Kolmogorov-Smirnov tests (Fig 2C). For pairs of chromosomes possessing a single CO between them, we compared the distance of late-HEI10 foci from the nearest chromosome end as a proportion of total chromosomal SC length. In total we analysed 664 and 514 single-CO bivalents from REC8 TTTT and REC8 DDDD plants, respectively. No significant differences in the position of single COs relative to chromosome ends were detected between genotypes (K-S Test, D = 0.04, *p* = 0.73). There was, however, a significant difference in the spacing of double-COs, by comparing the relative distance between late-HEI10 foci on paired chromosomes with two late-HEI10 foci, as a proportion of total chromosomal SC length. In total, we analysed 90 and 68 double-CO bivalents from REC8 TTTT and REC8 DDDD plants, respectively. Intriguingly, COs were on average closer together in REC8 TTTT cells (median relative inter-CO distance = 0.56) compared with REC8 DDDD cells (median relative inter-CO distance = 0.68, K-S test, D = 0.24, *p* = 0.02). Note that this result goes counter to expectation for evolved *A*. *arenosa*, which has more widely spaced COs than the neotetraploid [16].

From our pachytene measurements, we were also able to quantify the frequency of individual 4-chromosome units that were connected as either two pairs of synaptic bivalents or as a single synaptic quadrivalent (Fig 2D). A similar analysis was previously used to show that *A*. *arenosa* neo-polyploid pachytene cells contain a much higher relative frequency of synaptic quadrivalents than *A*. *arenosa* established tetraploids [16]. In REC8 TTTT pachytene cells, 75% of four-chromosome units (n = 300) were synapsed as pairs of bivalents and 25% (n = 100) were synapsed as quadrivalents. This is consistent with previously measured frequencies of synaptic bivalents and quadrivalents in *A*. *arenosa* established tetraploids [16]. The frequency of synaptic quadrivalents was slightly higher in the REC8 DDDD plants (30%, n = 94), although this difference was not statistically significant (Fisher’s exact test, *p* = 0.15).

In the subset of synaptic quadrivalents that contained a single SPS site and two COs, it was also possible to classify them as “*cis*” or “*trans*”. In *cis* quadrivalents the two COs occur on the same side of the SPS site (generating two bivalents at metaphase I), whilst in *trans* quadrivalents the two COs occur either side of the SPS site (generating one trivalent and one univalent at metaphase I). It was previously shown that neo-polyploid *A*. *arenosa* pachytene cells have a greater frequency of unfavourable *trans* configurations than established polyploid *A*. *arenosa* pachtyene cells [16]. Consistent with this, in both REC8 TTTT and REC8 DDDD pachytene cells, the relative frequency of *cis* configurations (for REC8 TTTT 96%, n = 69, for REC8 DDDD 97%, n = 58) vastly outnumbers the *trans* configurations (for REC8 TTTT 4%, n = 3, for REC8 DDDD 3%, n = 2), but there is no statistical difference between the two genotypes in these proportions (Fisher’s exact test, *p* = 1).

### Derived alleles of ASY1 and ASY3 quantitively alter REC8 allele-specific metaphase I phenotypes

Though the chromosome axes and cohesin complexes are distinct protein structures, REC8 is known to recruit the axis proteins and interact with them directly [32]. Two axis proteins, ASY1 and ASY3, also show strong evidence of having been under selection in tetraploid *A*. *arenosa* [10–12], and have been shown to have relevant functional effects on tetraploid meiosis [15]. Thus, we wished to test if there might be genetic interactions between the derived alleles of REC8, ASY1 and ASY3. To do this, we generated lines homozygous for the T or D alleles of all 3 genes (REC8, ASY1 and ASY3) in an otherwise tetraploid background. To achieve this, we crossed plants from the TBG population that were homozygous for the D alleles of ASY1 and ASY3 (ASY1 DDDD ASY3 DDDD) [15] with plants from the KOWA population that were homozygous for the D allele of REC8 (REC8 DDDD). The TBG x KOWA F_1_ plants were then inter-crossed to generate F_2_ and F_3_ populations of plants that segregated homozygotes for either the T or D alleles of REC8, ASY1 and ASY3 (S1 Fig). For brevity, we henceforth refer to the resultant REC8 TTTT ASY1 TTTT ASY3 TTTT and REC8 DDDD ASY1 DDDD ASY3 DDDD plants as RAA TTT and RAA DDD, respectively.

**Fig 3:**
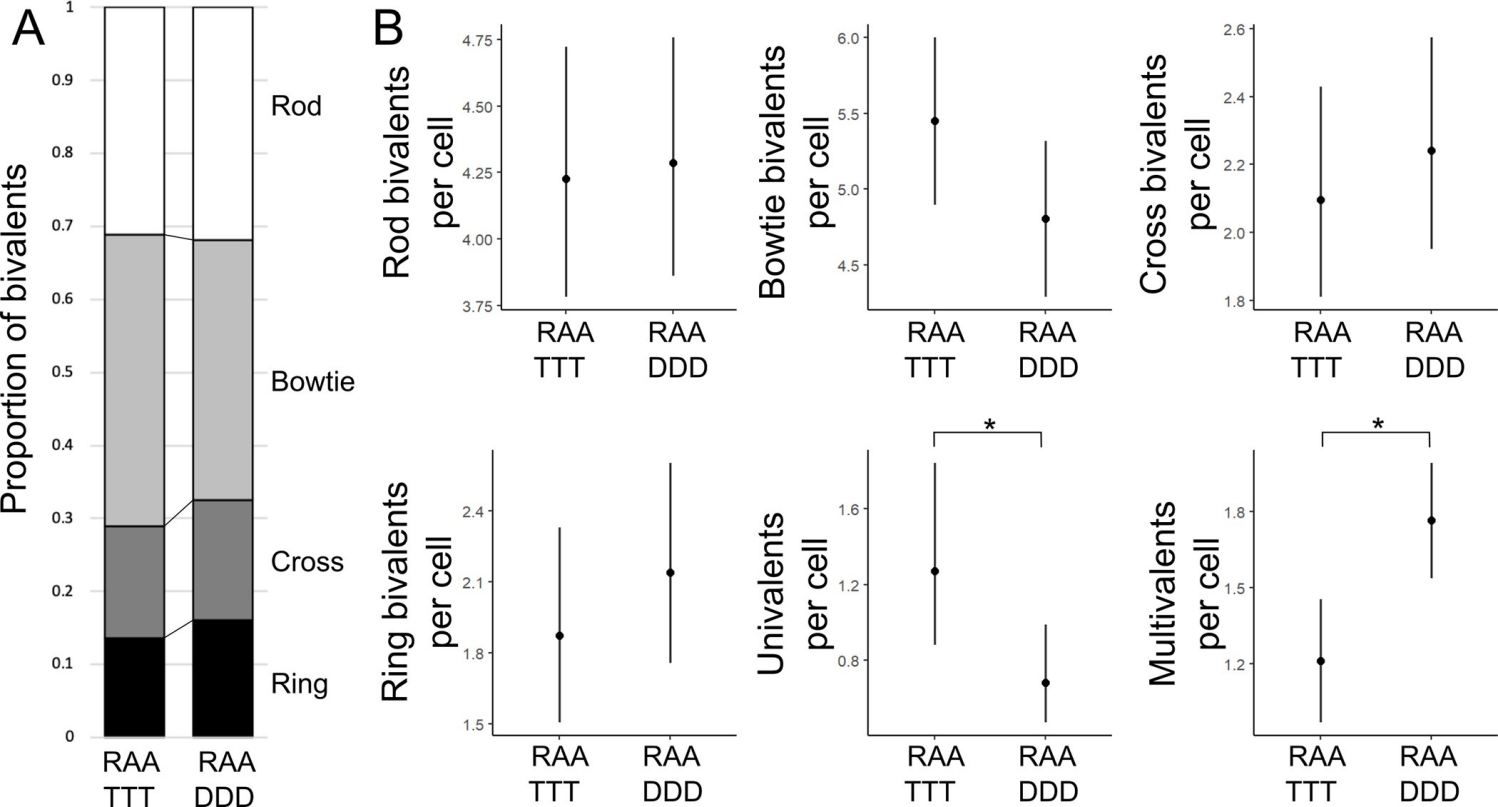
Analysis of RAA TTT and RAA DDD metaphase I cells. **(A)** Stacked bar chart showing the mean proportional frequency of different bivalent shapes in RAA TTT and RAA DDD metaphase I cells. **(B)** Plots showing the number of different chromosomal configurations per cell in RAA TTT and RAA DDD metaphase I cells. Dots indicate trait means and error bars 95% confidence intervals calculated from GLMM models. Significant between genotype *p* values are indicated: * *p* < 0.05.

We first quantified the frequency of different chromosomal configurations in meiotic metaphase I cells of RAA TTT and RAA DDD plants (Figs 3A and 3B and S4). In total, we imaged 102 cells from 4 RAA TTT plants and 108 cells from 5 RAA DDD plants (S4 Table). Regarding bivalent shape, no significant between-genotype differences were identified, however there was a trend for an increased frequency of bowtie bivalents in the RAA TTT cells compared with RAA DDD cells (LMM, 5.45, 95% CI [4.77, 6.13] vs. 4.80, 95% CI [4.19, 5.41], *p* = 0.14), which mirrored differences in bowtie bivalent frequencies in REC8 TTTT versus REC8 DDDD lines. Intriguingly, univalent frequencies were significantly increased in RAA TTT cells compared with RAA DDD cells (Poisson-GLMM, 1.27, 95% CI [0.88, 1.84] vs. 0.683, 95% CI [0.47, 0.99], *p* = 0.018), which was opposite to the difference observed for REC8 TTTT vs. REC8 DDDD. The RAA TTT cells also had significantly fewer multivalents compared with RAA DDD cells (LMM, 1.21, 95% CI [0.91, 1.51] vs. 1.77, 95% CI [1.50, 2.04], *p* = 0.0134), which mirrored the previously published effects of ASY1 and ASY3 T alleles on reducing metaphase I multivalent frequency [15], on which REC8 alleles had no effect (Table 1).

**Table 1:** Effect trends of ASY1, ASY3 and REC8 on several key meiotic traits.

Trait	ASY1* TTT / TDT	ASY3* TTT / TTD	ASY1+3* TTT / TDD	REC8 TTT / DTT	All 3 TTT / DDD	Comments
Rods (Tet>Dip)	**+60%**	(+22%)	**+50%**	(-10%)	=	ASY1 (and less so ASY3) increase rods, but, it appears, only when REC8 = T; could be antagonistic?
MV (Neo>Est)	**-29%**	(-17%)	(-24%)	=	**-32%**	**Only axis genes**, primarily ASY1
UV (Neo>Est)				**-71%**	**+86%**	**Antagonistic** effect
HEI10dist (Est>Neo)				**-18%**	(+22%)	**Antagonistic** effect
SClgth (Est<Neo)	**-11%**	(-3.5%)	(-4.6%)	(-6%)	(-5%)	T alleles of **all 3** correlate with shorter SC
SPS# (Est<Neo)	**-27%**	(-16%)	(-11%)	(-24%)	**-40%**	**All 3** contribute **additively** to reducing SPS#

Table notes: Effects of ASY1, ASY3 and REC8, or all three (column headings) on several key meiotic traits that differ between diploids and tetraploids (rods), or between neotetraploids and evolved tetraploids (all other traits) as shown below each trait name, where “Dip” = Diploid, “Tet” = Evolved Tetraploid, “Neo” = neo-tetraploid, “Est” = established tetraploid. “MV” = multivalent frequency, “UV” = univalent frequency, “HEI10dist” = single HEI10 focus distalisation, “SClgth” = SC length, “SPS#” = SPS site number. Homozygous genotypes for all three compared to yield single gene effects are given in headers for all three genes in each comparison as e.g. TTT vs DDD, showing REC8, ASY1, ASY3 in turn. Statistically significant differences are given in bold, and non-significant, but clearly trending ones in parentheses (as mentioned in the text, “trend” indicates a clear, but statistically not quite significant difference, e.g. p > 0.05, but near or below p = 0.1). Trend “direction” is given by a plus or minus with respect to the “T” (tetraploid) allele. In other words, “-”means that homozygotes for the T allele have a lower trait value relative to homozygotes for the D allele. “=“ indicates trait values for D and T are about equal. * Indicates that the left three columns are data trends from a separate, previously published experiment on ASY1 and ASY3 where REC8 was T [15].

### Derived alleles of REC8, ASY1 and ASY3 affect crossover patterning and synapsis in tetraploids

**Fig 4:**
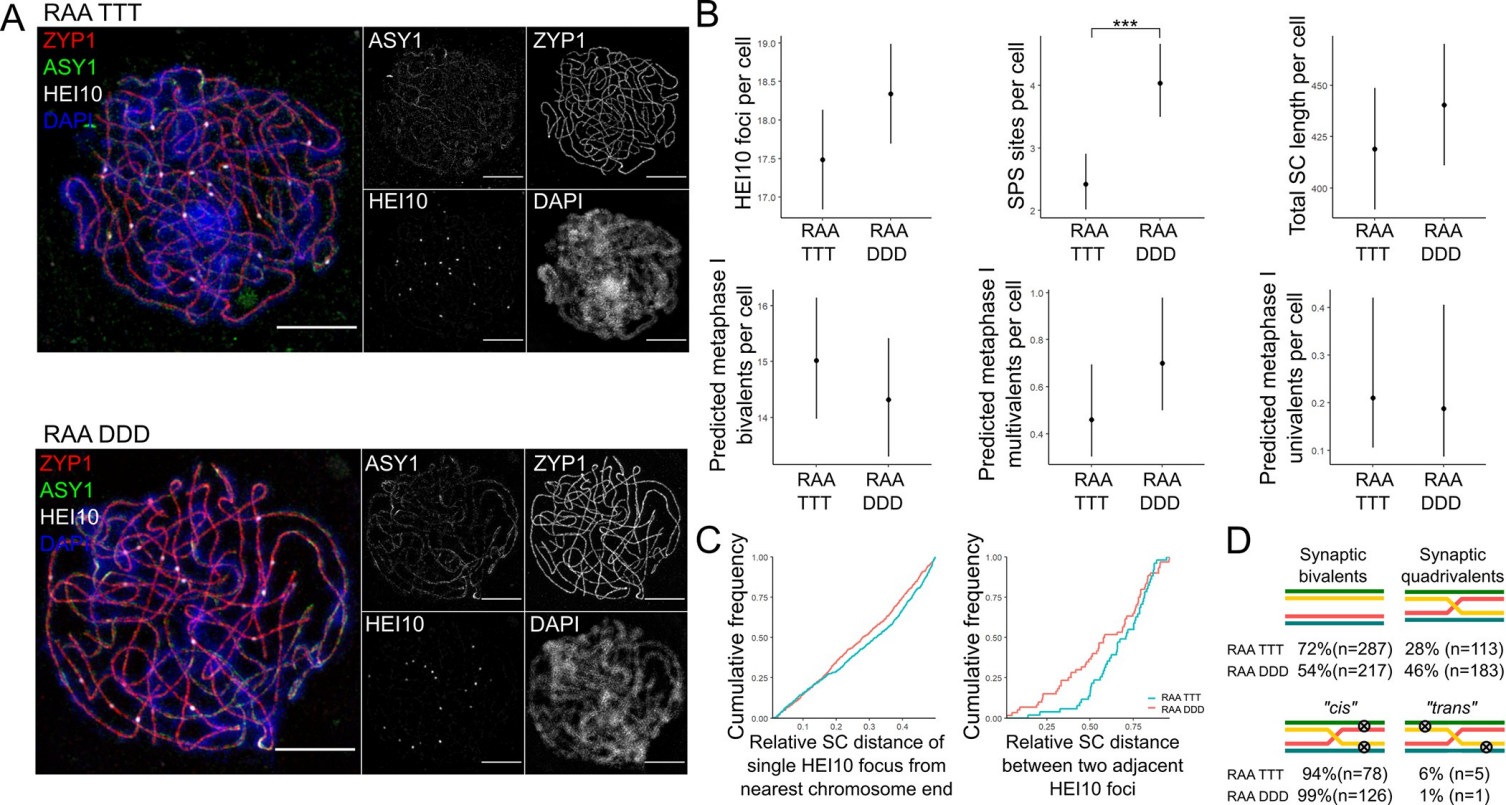
Analysis of RAA TTT and RAA DDD late-pachytene cells. **(A)** Example images of RAA TTT (top) and RAA DDD (bottom) late-pachytene cells imaged using 3D-SIM and labelled for ZYP1 (red), ASY1 (green), HEI10 (grey) and DAPI (blue). Maximum intensity projections of 3D images are presented. Scale bar = 5 μm. **(B)** Top row—plots showing the number of late-HEI10 foci (left), number of SPS sites (middle) and total SC length in μm (right) per cell in RAA TTT and DDD late-pachytene cells. Bottom row—plots showing the predicted number of metaphase I bivalents (left), multivalents (middle) and univalents (right) per cell based on the relative position of late-HEI10 foci and SPS sites along pachytene chromosomes in RAA TTT and DDD cells. Dots indicate trait means and error bars 95% confidence intervals calculated from GLMM models. Significant between genotype *p* values are indicated: *** *p* < 0.0005. **(C)** Cumulative frequency plots showing the distance of single late-HEI10 foci from the nearest chromosome end (left) and the distance between two late-HEI10 foci (right) in units of relative SC length in RAA TTT (blue) and RAA DDD (red) late-pachytene cells. **(D)** Frequency of different synaptic and crossover outcomes.

To further investigate the combined effects of derived alleles of REC8, ASY1 and ASY3 on CO patterning and synapsis, we imaged RAA TTT and RAA DDD late-pachytene cells labelled for ZYP1, HEI10, ASY1 and DAPI using 3D-SIM microscopy. In total, we imaged 50 cells from 5 RAA TTT plants and 50 cells from 5 RAA DDD plants (Fig 4A and S5 Table).

We found that RAA TTT plants had significantly fewer SPS sites per cell than RAA DDD plants (Poisson-GLMM, 2.42, 95% CI [2.02, 2.90] vs. 4.04, 95% CI [3.50, 4.65], *p =* 0.000012) and a trend for fewer COs per cell (LMM, 17.5, 95% CI [16.7, 18.2] vs. 18.3, 95% CI [17.6, 19.1], *p* = 0.1) (Fig 4B). The SPS reduction is similar to, but stronger than, that previously identified for plants that were segregating the T and D alleles of ASY1 and ASY3 (Table 1) [15], suggesting that for this trait, all three genes contribute additively to the evolved phenotype. The predicted metaphase I outcome data showed a trend that mirrored the metaphase I results, with RAA TTT cells tending to have fewer multivalents per cell than RAA DDD plants (Poisson-GLMM, 0.46, 95% CI [0.31, 0.69] vs. 0.7, 95% CI [0.50, 0.98], *p* = 0.12), while for predicted univalent / trivalents the difference was not significant (Poisson-GLMM, 0.21, 95% CI [0.11, 0.42] vs. 0.19, 95% CI [0.09, 0.40], *p* = 0.81).

Once again, we also analysed the relative distance of single-COs from bivalent ends and the relative distance between double-COs, and identified differences between the two genotypes (Fig 4C). In total, we analysed 699 RAA TTT and 655 RAA DDD single-CO bivalents, and 51 RAA TTT and 60 RAA DDD double-CO bivalents. The distribution of single COs was shifted significantly further away from the chromosome ends, in units of relative SC length, in the RAA TTT plants (median distance = 0.32) compared with the RAA DDD plants (median distance = 0.28, KS-test, D = 0.085, *p* = 0.016). This shift may explain the greater frequency of bowtie shaped metaphase I bivalents in the RAA TTT plants compared with the RAA DDD plants. No significant differences were detected in CO spacing between the two genotypes, although there was a trend for double-COs to be spaced slightly further apart in the RAA TTT plants (median distance = 0.71) compared with the RAA DDD plants (median distance = 0.58, KS-test, D = 0.24, p = 0.066), which is the opposite direction to the difference observed in REC8 TTTT versus REC8 DDDD plants.

A significant difference was also detected between genotypes in the relative frequency of synaptic bivalents and quadrivalents. The RAA TTT pachytene cells had a much greater frequency of synaptic bivalents (72%, n = 287) than RAA DDD cells (54%, n = 217), with RAA DDD cells therefore having a much greater frequency of synaptic quadrivalents (46%, n = 183) than RAA TTT cells (28%, n = 113) (Fisher’s exact test, *p* < 0.0001) (Fig 4D). Interestingly, however, there was a significantly greater frequency of unfavourable *trans* configurations within the RAA TTT synaptic quadrivalents (6%, n = 5) than in the RAA DDD cells (1%, n = 1) (Fisher’s exact test, p = 0.037), which is consistent with the RAA TTT cells exhibiting a greater frequency of metaphase univalents (Fig 4D).

## Discussion

As part of an ongoing effort to understand polygenic adaptation of meiosis after whole genome duplication in *Arabidopsis arenosa*, here we present a detailed cytogenetic analysis of established autopolyploid *A*. *arenosa* plants segregating derived (T) and ancestral (D) alleles of the meiotic genes *REC8*, *ASY1* and *ASY3*. We analyse effects observed in tetraploid plants carrying alternate alleles of *REC8* in an otherwise tetraploid genetic background, as well as plants carrying alternate alleles at all three genes. All three genes are part of a larger group of eight or so genes encoding interacting meiotic proteins showing strong evidence of having been targeted by natural selection during the evolution of autotetraploid *A*. *arenosa* [10–12,17]. We find evidence that there is a range of interactions among at least REC8, ASY1 and ASY3 derived alleles for different meiotic traits ranging from independent effects, to additive and in some cases even antagonistic interactions (Table 1). Our findings, in combination with prior discoveries in *A*. *arenosa*, provide information relevant to better understanding the perhaps non-linear adaptive walks that might occur during multigenic adaptation, and lead us to propose a multi-step evolutionary model for autopolyploid meiotic stabilisation, which we discuss below. An alternative, and not mutually exclusive view, is that REC8, which interacts directly or indirectly with almost all other meiotic proteins under selection in *A*. *arenosa* tetraploids, may act as a core node in an interacting set of proteins, and thus must co-evolve with all of them to continue to successfully coordinate their activities. In this latter model, REC8 would have been under selection not for its direct phenotypic effects, but rather for maintaining a functioning set of complex interactions among other evolving proteins. Both models could explain why REC8 has a strong signature of selection while having a relatively subtle effect on phenotype.

### T alleles of REC8, ASY1 and ASY3 genetically interact

A previous study comparing established tetraploid and neo-tetraploid *A*. *arenosa* plants (generated from colchicine-doubling of diploid plants) identified a number of phenotypic differences between these lines that likely explain the differences in meiotic fitness between the meiotically stable established tetraploids and meiotically unstable neo-tetraploids [16]. These differences can mostly be explained by increased effectiveness of crossover interference in the evolved tetraploids, resulting in altered synaptic and CO patterning behaviour in prophase I, which, ultimately, lead to lower frequency of harmful univalent and multivalent chromosomal configurations at metaphase I. Consistent with this, neo-tetraploids display a significantly higher frequency of both univalents and multivalents.

Strikingly, in this study we find that lines segregating the T or D alleles of REC8 display significant differences in the frequency of univalent chromosomes at metaphase I, with REC8 TTTT plants having fewer univalents than REC8 DDDD plants. This was not observed in a prior study of alternate alleles of the meiotic axis proteins ASY1 and ASY3 [15]. Surprisingly, in lines homozygous for the REC8 D as well the D alleles of ASY1 and ASY3 (instead of T alleles as above), the univalent phenotype is reversed, with RAA TTT plants possessing a greater frequency of univalents compared with RAA DDD plants (Table 1). In isolation, this observation might suggest that the RAA TTT plants have lower meiotic stability than the RAA DDD plants. However, the RAA TTT plants also display a significant reduction in multivalents (to which REC8 does not seem to contribute; Table 1) when compared with the RAA DDD plants, which would in turn lead to an *increase* in meiotic stability in the RAA TTT plants. The univalent and multivalent differences we observed in our metaphase I analysis are mirrored in the trends of predicted metaphase I fate of pachytene chromosomes based on the relative positions and frequencies of SPS sites and late-HEI10 foci. Taken together, these results suggest that the T alleles of REC8, ASY1 and ASY3 exert distinct, and in this case opposing, effects on stabilizing polyploid meiosis. Yet effects are not always antagonistic. For other traits, discussed below, effects are additive or independent (Table 1), hinting that pleiotropic multigenic adaptations can show complex interactions among alleles at different loci.

Trends in synapsis and CO patterning in late-prophase I cells are more consistent with additive, or at least non-antagonistic effects among the T alleles of all three genes (Table 1). Both the REC8 TTTT and RAA TTT plants also have fewer synaptic partner switch (SPS) sites and lower total synaptonemal complex (SC) length than the REC8 DDDD and RAA DDD plants, respectively. This mirrors differences between SPS number and SC length in established tetraploid and neo-tetraploid *A*. *arenosa* [16], with T alleles functioning to shift pachytene phenotypes closer towards those observed in more stable established tetraploids. In the case of SC length, T alleles of all three genes show effects (albeit at times subtle) in the same direction, and for SPS number, the three genes have what appear to be additive effects, with REC8 and ASY1 contributing to a similar extent, ASY3 less so, and all three genes together having a greater effect than any one of them alone (Table 1).

Interactions with additional genes under selection, which are as yet untested, will also be interesting, including the cohesin regulators SWI1 and PDS5, which both also show evidence of selection in tetraploid *A*. *arenosa* [10–12,17]. Intriguingly, it was recently shown in *S*. *cerevisiae* that Rec8 regulates chromosome axis length by modulating Pds5 abundance [42]. Pds5 depletion has also been shown to reduce chromosome axis length in *S*. *cerevisiae*, *S*. *pombe* and mice [43–45]. There are five orthologues of PDS5 in *A*. *thaliana* [46] and one of these, PDS5B, is under strong selection in tetraploid *A*. *arenosa* [10]. Whether or how derived alleles of these genes might interact to decrease axis length remains to be tested.

There was also a modest decrease in COs in the RAA TTT late-pachytene cells compared with the RAA DDD cells, but no difference was observed between REC8 TTTT and REC8 DDDD cells, suggesting either REC8 alleles do not differ in their effects on this trait, or that all three genes function synergistically. Mechanistically, the changes in CO number may be linked to the changes in SC length, as chromosome axis/SC length is known to influence CO frequency [38,47–49]. This decreased CO frequency, combined with the greater effect of ASY1 and ASY3 T alleles on decreasing SPS site frequency [15], likely explains why RAA TTT plants exhibit a decrease in metaphase I multivalents and an increase in univalents compared with RAA TTT plants.

Surprisingly, in REC8 TTTT plants the relative distance between double-COs was shorter than in REC8 DDDD plants, suggesting that the T allele of REC8 functions to weaken CO interference, which goes against the trend of the evolved polyploids, which seem to have more effective crossover interference than neopolyploids [16]. In RAA TTT plants, however, double-COs are spaced relatively further apart than in RAA DDD plants suggesting that the presence of the T alleles of ASY1 and ASY3 counteracts this REC8-T effect to promote stronger CO-interference. The involvement of the axis proteins is also consistent with recent experimental observations in *A*. *thaliana*, where CO positioning is sensitive to gene dosage of ASY1 and ASY3 (but not REC8) and CO interference is absent in *asy1* mutants [50]. For at least one trait, the proportion of rod bivalents, the effects of ASY1 and ASY3 seem to depend on a REC8-T allele being present, though REC8 genotype does not by itself affect this trait (Table 1).

### Suppression of univalents may represent an important first step in the evolution of stable autopolyploid meiosis

It was recently suggested that the sweep in REC8 might be one of the oldest of the tetraploid-adaptation sweeps in tetraploid *A*. *arenosa* [12]. Here, we find that the derived T allele of REC8 is associated with a reduced frequency of univalent chromosomal configurations at metaphase I in autopolyploids, likely positively contributing to meiotic stability. ASY1 and ASY3 derived alleles, oddly, seem to counteract this effect, while on the other hand reducing multivalents, another polyploid meiotic challenge to which REC8 alleles do not seem to contribute (Table 1). The derived REC8 allele also contributes quantitatively to several traits that ASY1 and ASY3 also contribute to, and in the same direction. We propose, based on the synthesis of these findings, that the T allele of REC8 might have been initially selected early in the evolutionary stabilisation of tetraploid *A*. *arenosa* lineages to promote the suppression of univalents, which are common in neo-polyploids [16]. Univalents pose a particularly serious problem for meiosis, and, hence, fertility, due to their extremely high likelihood of mis-segregating at anaphase I. In tetraploids, the effect of univalents on fertility is likely to be less pronounced compared with diploids, as the polyploid background acts as a buffer against the loss (or gain) or additional chromosome copies in aneuploid plants. Nonetheless, an increased likelihood of aneuploid offspring stemming from meiotic univalents will still lead to karyotypic instability over generations, negatively affecting overall population fitness. Therefore, the selection pressure to reduce the univalent frequency was likely very strong in *A*. *arenosa* neo-polyploids. The derived allele of REC8 likely solved this one issue, but either worsened others, or left them unsolved, which then later evolution of novel alleles at other interacting partners then helped mitigate, in an evolutionary “fine-tuning” process. As an alternative hypothesis, it may be that ASY1, and to a less extent ASY3, and/or perhaps other cohesin-interactors under selection in tetraploid *A*. *arenosa* are actually the prime drivers of many of the meiotic adaptations, while REC8 must co-evolve with its partners to maintain proper interactions, and that thus its own phenotypic effects are relatively subtle and diverse. This model would not explain why the REC8 sweep would be older, but here we caution that if there were successive sweeps on some of these genes, we can only see the most recent, meaning that depending on their history, ASY1 and ASY3 may have been under selection for longer than they appear, and may coincide with the timing of selection on REC8. Moreover, we note that these two models are not mutually exclusive, and both may contribute to different aspects of REC8 evolution.

Our first model is supported by the idea that likely the pressure to resolve univalent-associated issues will have been stronger than the pressure to resolve multivalent-associated issues. This is because, whilst multivalents are also prone to missegregation, the likelihood of missegregation is lower in multivalents than in univalents as, in some configurations, multivalent chromosomal associations will still lead to a high probability of balanced segregation [20]. Indeed, there are examples of some natural autotetraploid species that retain both a high frequency of multivalents and high levels of fertility [51], but we are not aware of reported cases where high univalent rates are tolerated in a sexually reproducing evolved tetraploid. Nonetheless, that multivalents still pose a problem is supported by their correlation with reduced fertility in many species [19], and thus we propose that, following the initial selection of the T allele of REC8 to resolve the more urgent univalent-issues, ASY1 and ASY3 were later selected to reduce the frequency of multivalents and strengthen CO interference and, in so doing, cause a partially undesirable but necessary uptick in the frequency of univalents. Likely this is tolerated merely because even though the increase relative to RAA DDD is significant, the overall univalent rate remains low in the RAA TTT plants.

Overall, our results hint that multigenic adaptation of meiosis in *A*. *arenosa* is also pleiotropic, and does not follow a linear accumulation of subtle additive effects, but rather represents a winding, and sometimes rough road up a novel fitness peak.

## Material and methods

### Plant material

Plants segregating the T and D alleles of REC8 were identified using PCR markers (see below) from the established autotetraploid KOWA accession of *A*. *arenosa*, collected from Kowary, Poland (50.76317N, 15.84389E). Plants segregating the T and D alleles of ASY1 and ASY3 were identified from the established autotetraploid TBG accession of *A*. *arenosa*, collected from Triberg, Germany (48.13972N, 8.23667E). A summary of the breeding strategy is shown in S1 Fig. In brief, two KOWA REC8 TTDD plants were identified and crossed and KOWA REC8 TTTT, TxD and DDDD plants identified in the F_1_ generation were used for cytological investigation. Two TBG ASY1 TDDD ASY3 TDDD plants were also identified and crossed and TBG ASY1 DDDD ASY3 DDDD plants were identified in the F_1_ population. TBG ASY1 DDDD ASY3 DDDD plants were then crossed with KOWA REC8 DDDD plants and progeny were backcrossed for several generations to generate REC8 TTTT ASY1 TTTT ASY3 TTTT (RAA TTT) and REC8 DDDD ASY1 DDDD ASY3 DDDD (RAA DDD) plants that were used for cytological investigation. All plants were grown together in the same controlled environment room with 16 h of light (125 mMol cool white) at 20°C and 8 h of dark at 16°C.

### Genotyping of REC8, ASY1 and ASY3

DNA extraction and genotyping of ASY1 and ASY3 using CAPS markers was as described in [15]. Genotyping for REC8 diploid (D) versus tetraploid (T) alleles was carried out using a KASP marker and further confirmed using a CAPS marker and Sanger sequencing. The KASP genotyping was carried out as described in [52] with the following changes: 35 cycles of amplification were used, and the 384 plate was read on a PHERAstar plate reader (PHERAstar FSX Microplate Reader for HTS | BMG LABTECH). KASP primers for REC8 were F1_FAM 5’ GAAGGTGACCAAGTTCATGCTTTCATGGGAACTACAGGAGA 3’, F1_VIC 5’ GAAGGTCGGAGTCAACGGATTTTCATGGGAACTACAGGAGG 3’ (where GAAGGTGACCAAGTTCATGCT and GAAGGTCGGAGTCAACGGATT are the FAM and VIC tails, respectively) and common primer R1 5’ TAATTGAAGCTGCTCCACGT 3’. These were designed around the D versus T SNP allele (see S3 Fig). Primers for REC8 CAPS marker were F 5’ CGTGCGAATATGCCACCTA 3’ and R 5’ TCTTGGTGAGGAAGTTGGC 3’ and the PCR amplicon was digested with Alw26I (BsmAI) (10 U/μL) (Thermofisher) at 37°C for 1.5 hours. The diploid allele was cut twice and thus gave products of 527bp, 220bp and 115bp; whereas the tetraploid allele was only cut once and thus gave two products of 747bp and 115bp. (see Alw26 restriction sites in S3 Fig). Genotyping for ASY3 was initiated with a KASP marker using the following primers: R1_FAM 5’ GAAGGTGACCAAGTTCATGCTTTCCCCTTTGTTTCTTTGGC 3’, R1_VIC 5’ GAAGGTCGGAGTCAACGGATTTTCCCCTTTGTTTCTTTGGT 3’ and common primer F1 5’ ATTGGCACTGCTATGAATTC 3’. The assay for this marker was as described for REC8 KASP. This KASP marker for ASY3 identified DDDD genotypes but could not distinguish DxT from TTTT genotypes. Thus, further genotyping was carried out by the ASY3 CAPS marker.

### Metaphase I cytology

For the preparation of DAPI stained metaphase I spreads from *A*. *arenosa* we followed the protocol described in [53], with minor modifications. First, inflorescences were fixed in 3:1 ethanol:glacial acetic acid. Buds of the correct size (approx. diameter 0.9mm) were removed from the fixed inflorescences and incubated in 300 μl enzyme mix (0.3% cellulase, 0.3% pectolyase in 10 mM citrate buffer) in a moist chamber for 90 minutes. For each slide, two digested buds were transferred into 2 μl of 80% acetic acid on the slide surface and macerated briefly with a brass rod. An extra 20 μl of acetic acid was then added to the slide and the slide was placed on a 45°C hot block for 30 seconds whilst a mounted needle was used to stir the liquid droplet. 2 x 200 μl of 3:1 ethanol:acetic acid was then added and the slide was dried using a hairdryer. Slides were mounted in 7 μL 1 μg/mL DAPI in Vectashield mounting medium (Vector Laboratories) and imaged using a Zeiss Axio Imager epifluorescence microscope. All metaphase I images are freely available via the ETH Research Collection under DOI: 10.3929/ethz-b-000542141.

### Immunocytology

For the preparation of immunostained pachytene spreads for 3D-SIM imaging we followed the protocol described in [54]. First, anthers containing meiocytes of the desired stages were dissected from fresh floral buds. Anthers were then macerated in 10 μl digestion medium on a No. 1.5 coverslip (Marienfeld) using a brass rod and incubated at 37°C in a moist chamber for 4 minutes. 10 μl of 2% Lipsol (SciLabware) was then added to the coverslip and spread out using a mounted needle, before adding 20 μl of 4% paraformaldehyde (pH 8) and leaving the coverslip to dry for 3 hours. The coverslip was then blocked in 0.3% bovine serum albumin in 1 x phosphate buffered saline (PBS) solution for 15 minutes at room temperature. 50 μl of primary antibody was added to the coverslip and incubated overnight at 4°C. 50 μl of secondary antibody was added to the coverslip and incubated at 37°C for 2 hours. The coverslips were washed in 1 x PBS before and after each antibody addition. Finally, coverslips were incubated in 10 μg/mL DAPI for 5 min and mounted on a slide in 7 μL Vectashield (Vector Laboratories). The following primary antibodies were used at 1:500 dilutions: anti-ASY1 (guinea pig), anti-ZYP1 (rat), and anti-HEI10 (rabbit). The following secondary antibodies were used at 1:200 dilutions: anti-guinea pig Alexa Fluor 488 (Thermo Fisher), anti-rat Alexa Fluor 555 (F(ab′)2 fragment, Abcam) and anti-rabbit Alexa Fluor 647 (F(ab’)2 fragment, Thermo Fisher). Cells were imaged using a Zeiss Elyra PS1 microscope in 3D-SIM mode. 3D measurements of SC length and CO and SPS positions were performed using the Simple Neurite Tracer plugin to ImageJ [40]. All immunocytological images are freely available via the ETH Research Collection under DOI: 10.3929/ethz-b-000542141.

### Statistical analysis

All statistical analyses were performed in R (Version 1.2.5019). As described previously [15], generalized linear mixed models (GLMMs) and linear mixed-effect models (LMMs), and associated p values, were obtained using the glmer() and lmer() functions of the lme4 R package. Poisson-GLMMs were utilised for count data and LMMs for continuous data, as appropriate. LMMs were also substituted for Poisson-GLMMs in the event of a singular fit. Predicted means, standard errors and 95% confidence intervals were obtained using the lsmeans() function. Kolmogorov-Smirnov tests, Kruskal Wallace tests and posthoc Dunns tests were performed using the functionsks.test(), Kruskal.test() and dunnTest(), respectively.

## Supporting information

S1 Fig Overview of breeding strategy. Lines used for analysis are labelled in red. Dotted line indicates several generations of backcrossing.
(TIFF)

S2 Fig Normalised analysis of REC8 TTTT, TxD and DDDD metaphase I cells. Plots showing the percentage of chromosomes contained in different chromosomal configurations per cell (normalised against the total number of scorable chromosomes per cell) in REC8 TTTT, TxD and DDDD metaphase I cells. Dots indicate trait means and error bars 95% confidence intervals calculated from GLMM models. Significant between genotype p values are indicated: * p < 0.05.
(TIFF)

S3 Fig Quantitative 3D-SIM imaging of late-pachytene cells. **(A)** Example image of a REC8 TTTT late-pachytene cell imaged using 3D-SIM and labelled for ZYP1 (red), ASY1 (green), HEI10 (grey) and DAPI (blue). Maximum intensity projections of 3D images are presented. Yellow boxes highlight the positions of SPS sites and magnified images of these regions are shown. A 3D model of this cell, generated using the Simple Neurite Tracer plugin to ImageJ, is also shown, with synapsed pairs of chromosomes labelled in different colours and CO sites marked by green spheres. Scale bar = 5 μm. **(B)** 3D models of the two synaptic quadrivalents from the cell in (A) are shown. Cartoon diagrams depicting each component chromosome in a different colour and CO sites as black circles are shown to demonstrate how chromosomes switch their synaptic partner. A straightened and scaled version of each cartoon diagram is also shown and quantitative measurements of CO and SPS site positions are indicated. The predicted metaphase I outcome of each synaptic quadrivalent is also shown.
(TIFF)

S4 Fig Normalised analysis of RAA TTT and RAA DDD metaphase I cells. Plots showing the percentage of chromosomes contained in different chromosomal configurations per cell (normalised against the total number of scorable chromosomes per cell) in RAA TTT and RAA DDD metaphase I cells. Dots indicate trait means and error bars 95% confidence intervals calculated from GLMM models. Significant between genotype p values are indicated: * p < 0.05, ** p < 0.005.
(TIFF)

S5 Fig Alignment of REC8 fragment used for genotyping. The top sequence is an *Arabidopsis arenosa* tetraploid sequence obtained from a draft assembly [12]. The other two sequences were obtained from Sanger sequencing of PCR product of DNA from two KOWA plants using the CAPS primers. In yellow: REC8 primers for CAPS marker; in turquoise: D versus T SNP allele for CAPS genotyping; boxed up and clear: REC8 KASP primers; and boxed up and grey: Alw26 restriction sites.
(TIFF)

S1 Table Chromosomal configurations scored from REC8 TTTT, TxD and DDDD metaphase I cells. (CSV)

S2 Table Summary of p-values from non-parametric significance tests of metaphase I chromosomal configuration data. (CSV)

S3 Table Measurements from REC8 TTTT and REC8 DDDD late-pachytene cells. (CSV)

S4 Table Chromosomal configurations scored from RAA TTT (4xTTTT) and RAA DDD (4xDDDD) metaphase I cells. (CSV)

S5 Table Measurements from RAA TTT and RAA DDD late-pachytene cells. (CSV)
