# Supplementary figures and images for: The meiotic cohesin subunit REC8 contributes to multigenic adaptive evolution of autopolyploid meiosis in Arabidopsis arenosa
Source: PLoS Genet. 2022 Jul 13;18(7):e1010304. doi: 10.1371/journal.pgen.1010304 (PMC9312919; doi:10.1371/journal.pgen.1010304)

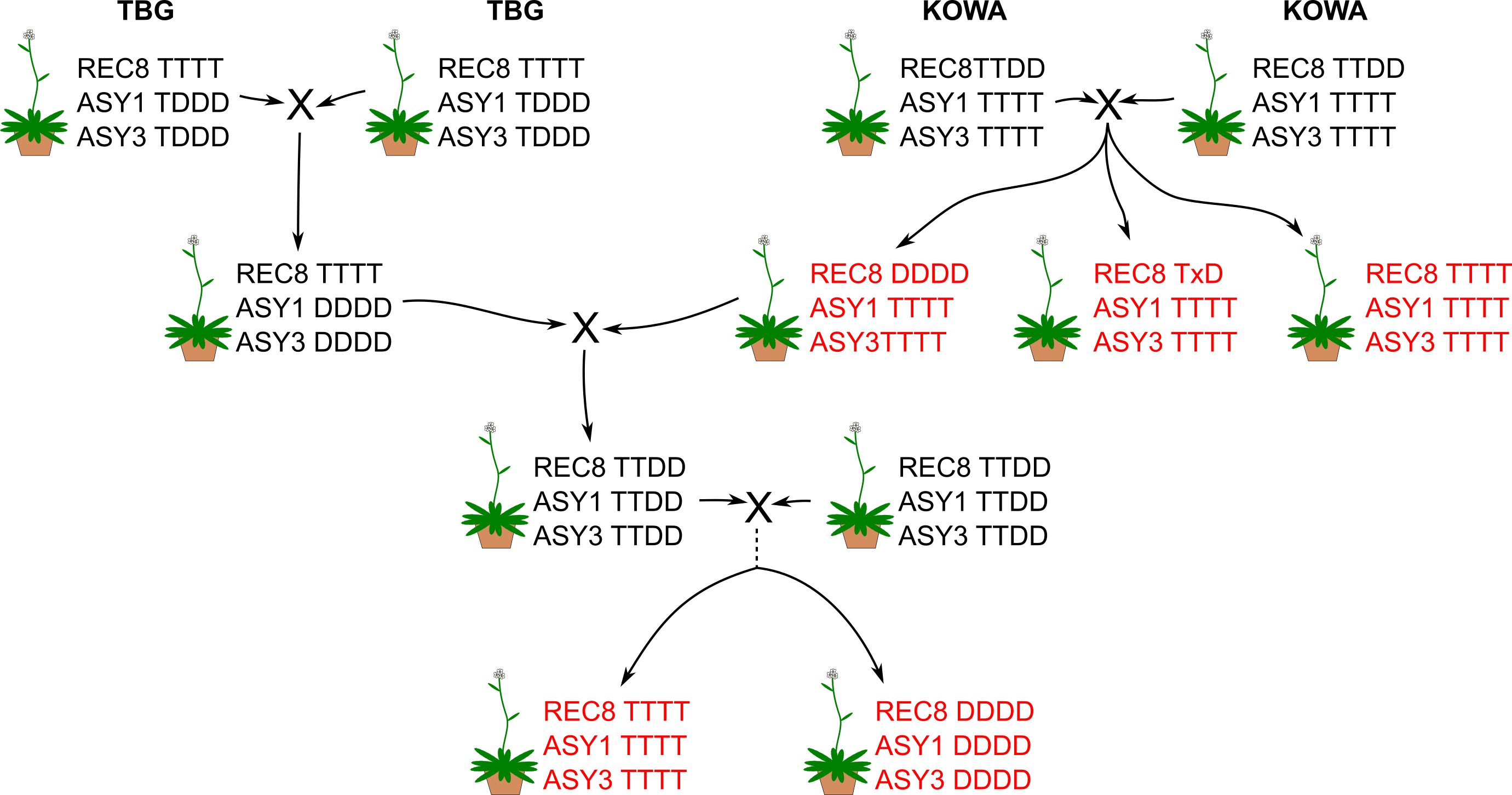

Supplement: S1 Fig — Lines used for analysis are labelled in red. Dotted line indicates several generations of backcrossing. (TIFF) [file pgen.1010304.s001.tiff]

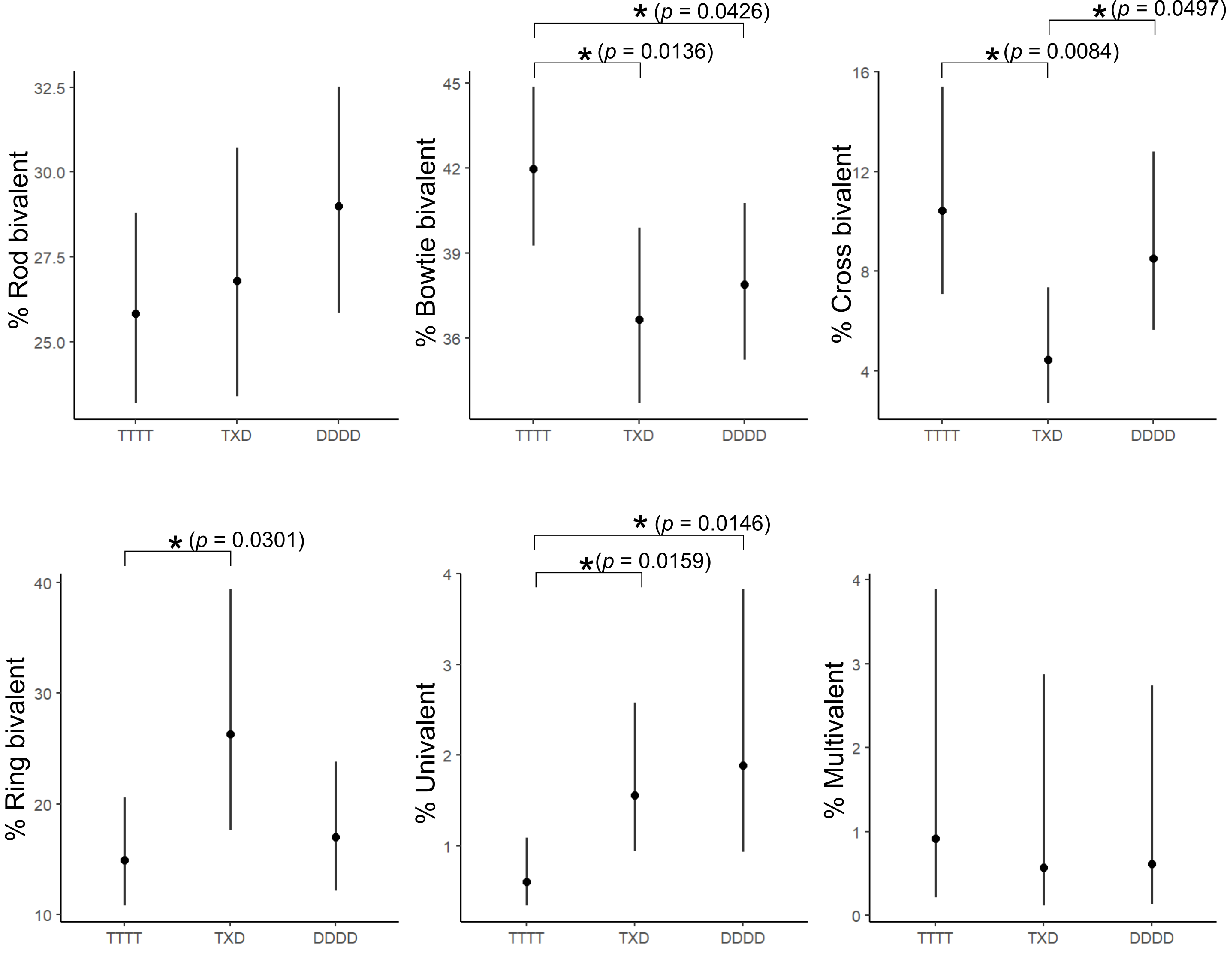

Supplement: S2 Fig — Plots showing the percentage of chromosomes contained in different chromosomal configurations per cell (normalised against the total number of scorable chromosomes per cell) in REC8 TTTT, TxD and DDDD metaphase I cells. Dots indicate trait means and error bars 95% confidence intervals calculated from GLMM models. Significant between genotype p values are indicated: * p < 0.05. (TIFF) [file pgen.1010304.s002.tiff]

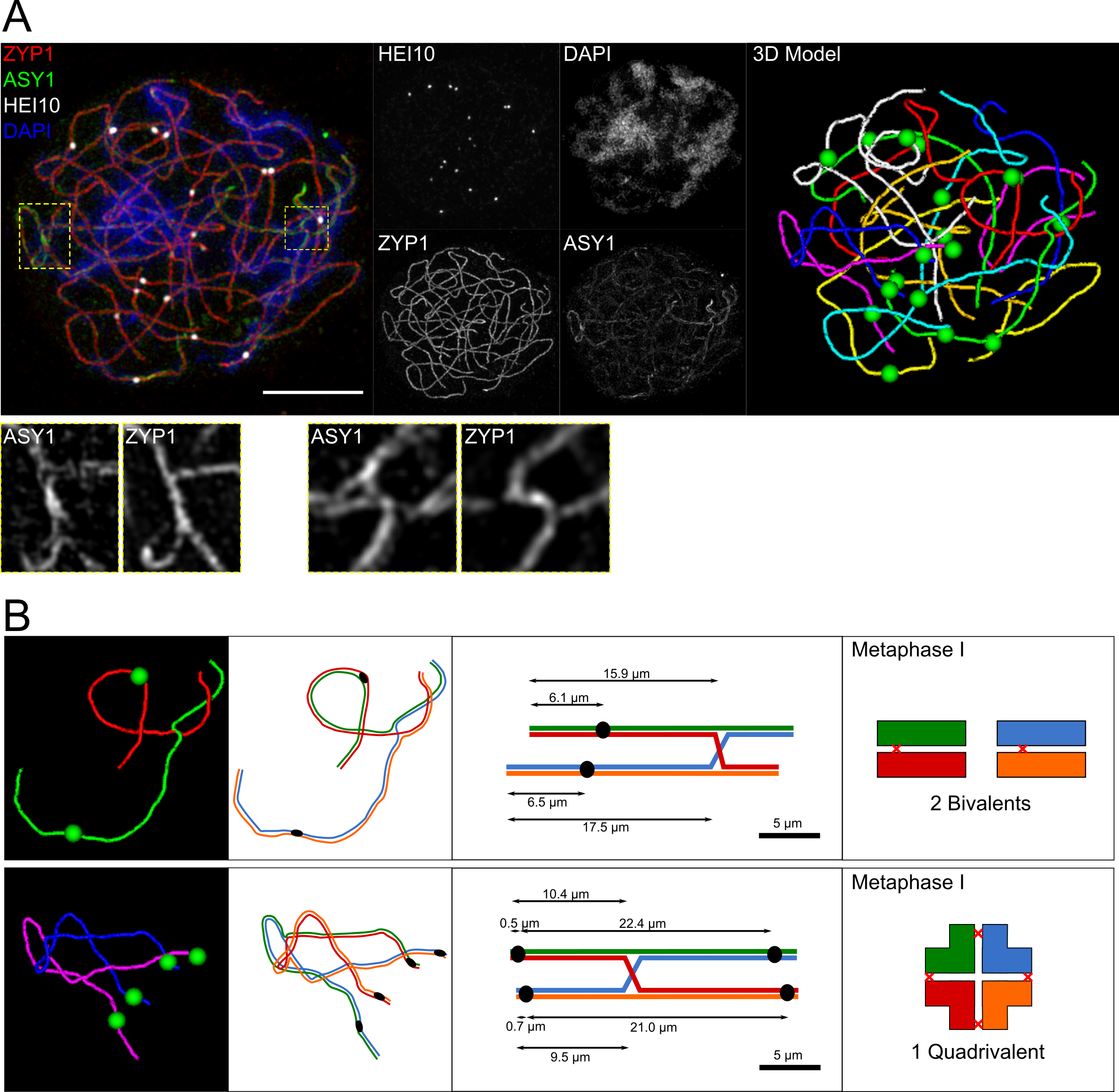

Supplement: S3 Fig — (A) Example image of a REC8 TTTT late-pachytene cell imaged using 3D-SIM and labelled for ZYP1 (red), ASY1 (green), HEI10 (grey) and DAPI (blue). Maximum intensity projections of 3D images are presented. Yellow boxes highlight the positions of SPS sites and magnified images of these regions are shown. A 3D model of this cell, generated using the Simple Neurite Tracer plugin to ImageJ, is also shown, with synapsed pairs of chromosomes labelled in different colours and CO sites marked by green spheres. Scale bar = 5 μm. (B) 3D models of the two synaptic quadrivalents from the cell in (A) are shown. Cartoon diagrams depicting each component chromosome in a different colour and CO sites as black circles are shown to demonstrate how chromosomes switch their synaptic partner. A straightened and scaled version of each cartoon diagram is also shown and quantitative measurements of CO and SPS site positions are indicated. The predicted metaphase I outcome of each synaptic quadrivalent is also shown. (TIFF) [file pgen.1010304.s003.tiff]

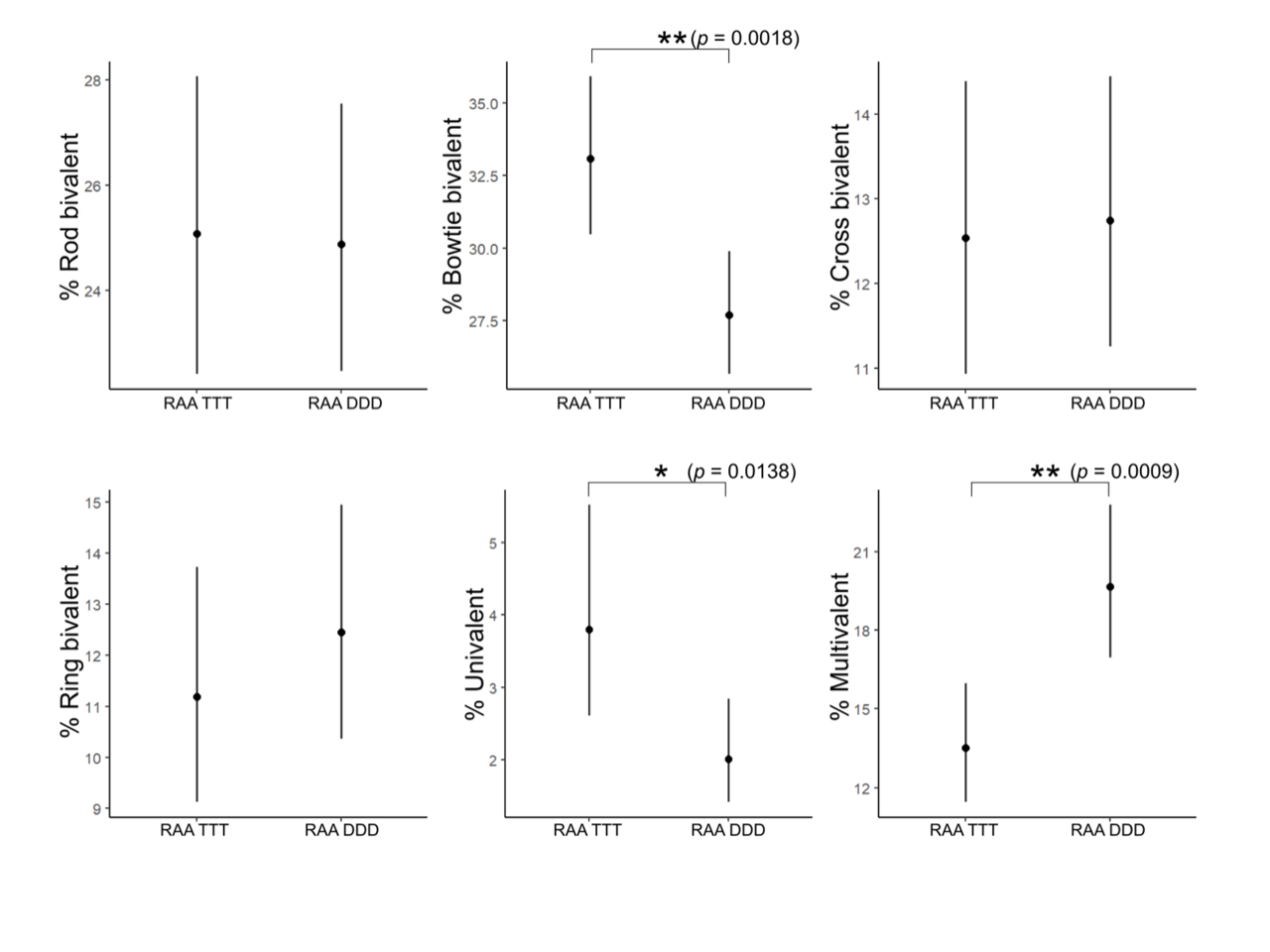

Supplement: S4 Fig — Plots showing the percentage of chromosomes contained in different chromosomal configurations per cell (normalised against the total number of scorable chromosomes per cell) in RAA TTT and RAA DDD metaphase I cells. Dots indicate trait means and error bars 95% confidence intervals calculated from GLMM models. Significant between genotype p values are indicated: * p < 0.05, ** p < 0.005. (TIFF) [file pgen.1010304.s004.tiff]

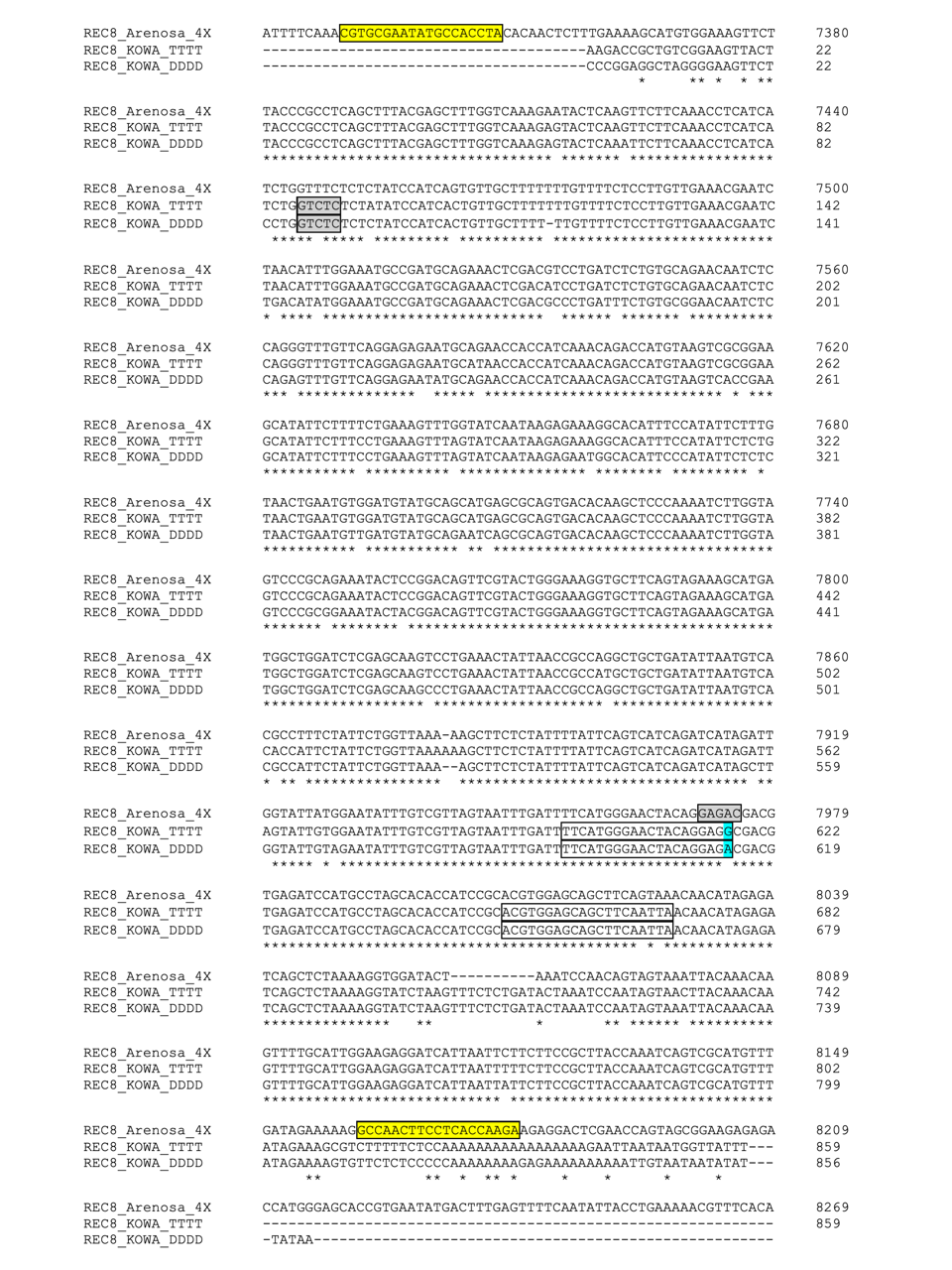

Supplement: S5 Fig — The top sequence is an Arabidopsis arenosa tetraploid sequence obtained from a draft assembly [12]. The other two sequences were obtained from Sanger sequencing of PCR product of DNA from two KOWA plants using the CAPS primers. In yellow: REC8 primers for CAPS marker; in turquoise: D versus T SNP allele for CAPS genotyping; boxed up and clear: REC8 KASP primers; and boxed up and grey: Alw26 restriction sites. (TIFF) [file pgen.1010304.s005.tiff]
